# The Investigation of the Sensitivity of the Compliance to the Shape of the Spot in Welded Thermoplastic Single-Lap Shear (SLS) Joints

**DOI:** 10.3390/ma19102016

**Published:** 2026-05-12

**Authors:** Eva T. B. Smeets, Calvin D. Rans, René Alderliesten, Irene Fernandez Villegas

**Affiliations:** Department of Aerospace Structures and Materials, Faculty of Aerospace Engineering, Delft University of Technology, Kluyverweg 1, 2629 HS Delft, The Netherlands; c.d.rans@tudelft.nl (C.D.R.); r.c.alderliesten@tudelft.nl (R.A.); i.fernandezvillegas@tudelft.nl (I.F.V.)

**Keywords:** ultrasonic welding, damage characterization, structural testing, numerical modelling

## Abstract

To ensure safety in structural design, a method to quantify the damage in thermoplastic ultrasonic single-spot-welded Single-Lap Shear (SLS) joints is needed. This paper investigates whether detailed knowledge regarding the shape of the weld is required when using the global compliance to quantify damage. A finite element model using cohesive zone elements is developed in Abaqus to simulate single-spot SLS specimens with varying weld areas, aspect ratios, and damage growth directions, covering damage levels from 0 to 90% of the initial weld area. For each configuration, the relationship between intact weld area and global compliance is evaluated, and the numerical trends are compared to previously published experimental data from similar joints. The results show that weld size and damage growth direction have negligible influence on the relationship between global compliance and weld area, and that weld shape is also insignificant as long as the aspect ratio remains within a practical range; only very elongated welds with an aspect ratio over 4.4, which are unlikely in production, deviate significantly. Global compliance can be used as a reliable indicator of damage in single-spot ultrasonic welds that is insensitive to weld shape. This enables simplified in situ damage monitoring and reduces the need for detailed geometric characterisation during mechanical testing.

## 1. Introduction

In the aerospace industry, safety is a crucial factor when developing new materials and techniques. To ensure structural safety, a damage tolerance or a safe-life approach can be used [1]. For both of these strategies, quantification of damage, both during operation and development, is needed. With traditionally used metal materials, the damage modes are such that the damage is visible on the surface of the structure. Today, Fibre Reinforced Polymers (FRPs) are gaining more interest. These materials pose new challenges due to the more complex damage modes. Significant damage can be present in a structure without any visible signs. With thermoplastic ultrasonic welds, the most common damage mode for strong welds is inter-laminar failure, which is one layer above or below the weld itself [2]. Weaker welds exhibit either interfacial failure or a combination of inter-laminar and interfacial failure. In all of these cases, the damage is located at the welded interface. When considering continuously welded joints in an experimental context, the weld spans the whole width and is visible from the edges, and the damage can be localised with visual inspection methods. However, in spot welds, the interface cannot be seen; thus, an in situ inspection method that does not rely on visibility is needed.

The study reported by Smeets et al. [3] investigated the use of Digital Image Correlation (DIC) measurements to achieve such an in situ technique for damage quantification. Due to variations in the welding process, this publication included circular welds and more irregularly shaped welds. It was shown that the comparison between the local weld area and the global specimen compliance is in correspondence. This raises the question whether the global compliance provides sufficient information to adequately represent the damage state of the interface without the need for a detailed inspection of the specific weld shape. If it can be proven that the relationship between compliance and area is indeed insensitive to a change in weld shape, it could simplify the in situ damage quantification in joints where the interface cannot be directly observed from the outside of the specimens, since the damage state of the weld can then be described with a single parameter.

Ultrasonic Welding (USW) was first applied to metal materials in the 1950s [4], expanded to plastics in the 1960s, and to plastic composites in the late 1980s [5]. Currently, the most widespread application is plastic welding of packaging [5]. Plastic welding is rarely applied in load-bearing structures, and thus, research regarding the structural performance is nonexistent to the author’s knowledge. USW of metal adherends is commonly used in battery manufacturing and lightweight automotive parts [6]. For metal USW, most of the research focuses on the influence of the processing parameters on the joint performance [7,8]. The research regarding fatigue performance focuses on the damage mechanisms and the influence of processing parameters on the fatigue life [9,10]. Investigations of fatigue damage mechanisms have shown that USW joints exhibit either nugget pull-out [11] or interfacial failure [9,10,12] when subjected to a high cyclic load. In the case of low cyclic loads, these studies always observed through-thickness cracking due to micro-cracking of the adherends near the edges of the welds during manufacturing [12]. The current knowledge is limited to the relation between the processing parameters and the resulting weld shape. How the shape of the weld influences the fatigue life is unknown.

Since the application of ultrasonic welding to thermoplastic composite materials is more recent, research efforts have been mostly focused on the influence of varying processing parameters on weld quality and mechanical strength. For example, the effect of changing the vibration amplitude and pressure, and other welding parameters [13,14,15,16,17,18,19], adherend thickness [20,21], interface crystallinity [22,23], weld quality assessment [24], energy directors [25], and the effect of different matrix materials [26] has been studied. It should also be noted that contamination of the welded area, for example, with the release agent left from plate manufacturing, has a significant influence on the quality and mechanical properties of the resulting joint [27]. The eco-mechanical efficiency and life cycle of ultrasonically welded components have also been investigated [28]. More recently, the fatigue failure mechanisms of thermoplastic joints have been investigated [29], as well as for hybrid aluminium–FRP joints [30]. Sioutis and Tserpes developed a mixed-mode fatigue growth model for co-consolidated thermoplastics that could translate to welded specimens [31]. Völkerink et al. looked into the certification of composite welded joints using a virtual testing approach [32]. Similar to metal ultrasonic spot welds, the knowledge regarding the shape of thermoplastic ultrasonic spot welds is limited to their relation to the processing parameters. The influence of the shape of the spot weld on the mechanical properties of the joint is not known.

With this in mind, this study aims to investigate whether global compliance sufficiently captures the damage state in a Single-Lap Shear (SLS) joint without any knowledge regarding the shape of the weld. The limits of such a correlation were also investigated. Due to the challenges in controlling the precise shape and size of the spot weld and the limited availability of experimental data, a series of Finite Element Analysis (FEA) was used to investigate the correlation. For each of these pristine spots, several damaged states with damage percentages between 0 and 90% of the initial area were analysed. Finally, the results were compared to previously published experimental data from the publication by [3].

## 2. Materials and Methods

To investigate the influence of the weld shape on the relationship between its area and global compliance, an SLS specimen configuration was analysed with different weld shapes. The global compliance is calculated using the static failure displacement and the corresponding load. Three aspects of the weld shape were considered: the total welded area, the initial aspect ratio, and whether the damage develops symmetrical or asymmetrical. To allow control over the precise weld shape and size, a series of static Finite Element (FE) analyses were performed in which damage was increased by deleting elements at the edges of the weld. The weld sizes were chosen based on the welds that were observed during the manufacturing process by Smeets et al. [3] using a sonotrode with a diameter of 20 mm. The details regarding the welding process parameters and other manufacturing specifications are shown by Smeets et al. [3]. The aspect ratios represent the range reported in the same study, with the addition of smaller and larger values to explore the limits of the current hypothesis. For the purpose of this study, compliance is defined as the ratio between the applied displacement and the resulting reaction force.

A description of the base configuration is included in Section 2.1, the parameters for the spot welds are detailed in Section 2.2. Section 2.3 describes the method used for damage incrementation and in Section 2.4 the FE models and boundary conditions are described.

### 2.1. Single-Lap Shear Specimens and Material

**Figure 1:**
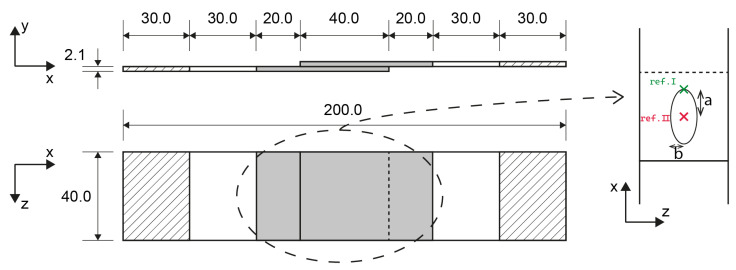
Single lap shear configuration with the dimensions of the spot and the reference points for asymmetric or one-sided damage growth (ref. I) and symmetric or two-sided damage growth (ref. II).

Figure 1 shows the configuration of the SLS specimens analysed with the FE models. The configuration was based on ASTM standard D5686 [33]. To facilitate the validation with the experimental data [3], the overlap width and length were both increased to 40 mm. The hatched areas at both ends of the specimen indicate the region where the boundary conditions are applied. To gradually introduce the load, the choice was made to apply the boundary conditions over the entire area instead of only using the outer edge of the specimen. The grey shaded area in the centre of the specimen is the region of interest. The material used for the adherends was a standard modulus carbon unidirectional tape with a low-melt polyaryletherketone (LMPAEK) matrix. The parameters are shown in Table 1.

### 2.2. Spot Weld Parameters

Different spot sizes and shapes were tested. The spot welds had an aspect ratio (*b*/*a*) which varied between 0.23 and 4.43, and an initial weld area between 117 mm^2^ and 420 mm^2^. For each of these shapes, there was one analysis with the undamaged spot and two sets of nine analyses with a damaged state. In one set, the damage was symmetrical on the top and the bottom of the spot, and in the second set, the damage was only on the top of the specimen. The details of all different weld dimensions are presented in the test matrix in Table 2, the definition of the ellipse dimensions and the reference points are illustrated in Figure 1.

**Table 2:** Spot weld test matrix showing the weld geometries (pristine area, aspect ratio, and damage sizes) and the corresponding sketch and results.

Pristine Weld Area [mm]	Aspect Ratio [−]	$\frac{b\;[\mathrm{mm}]}{a\;[\mathrm{mm}]}$	Damage Sizes [%]		Figure Symbols			
			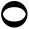	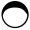	Figure 2	Figure 6	Figure 7	Figure 8
117	-	-	-	-	-	-	-	-
205	0.23	3.5/15.5	10–90	10–90	-	-	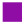	-
420	-	-	-	-	-	-	-	-
117	0.41	3.5/8.5	10–90	10–90	Figure 2c	-	-	-
205	-	-	-	-		-	-	-
420	0.41	7/17	10–90	10–90		-	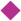	-
117	-	-	-	-	-	-	-	-
205	-	-	-	-	-	-	-	-
420	0.61	8.5/14	10–90	10–90	-	-	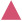	-
117	1	5.5/5.5	10–90	10–90	Figure 2a,b	-	-	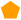
205	1	7.5/7.5	10–90	10–90		-	-	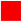
420	1	11/11	10–90	10–90		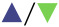	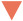	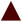
117	-	-	-	-	-	-	-	-
205	-	-	-	-	-	-	-	-
420	1.65	14/8.5	10–90	10–90	-	-	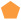	-
117	2.43	8.5/3.5	10–90	10–90	Figure 2d	-	-	-
205	-	-	-	-		-	-	-
420	2.43	17/7	10–90	10–90		-	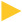	-
117	-	-	-	-	-	-	-	-
205	4.43	15.5/3.5	10–90	10–90	-	-	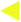	-
420	-	-	-	-	-	-	-	-

**Figure 2:**
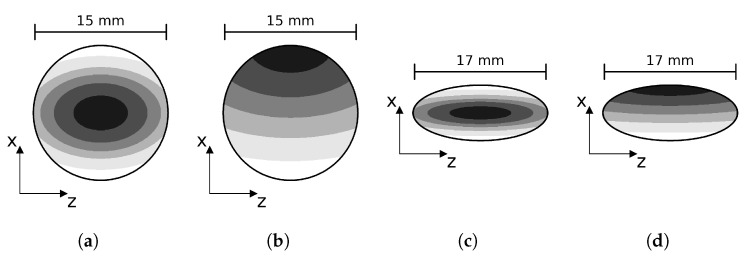
Visualisation of the damage incrementation strategy for different test cases. (**a**) Aspect ratio = *b*/*a* = 1, two-sided growth. (**b**) Aspect ratio = 1, one-sided growth. (**c**) Aspect ratio = 3.5/8.5, two-sided growth. (**d**) Aspect ratio = 3.5/8.5, one-sided growth.

### 2.3. Damage Incrementation Strategy

To determine the different damaged scenarios, a weighted distance $d_{w}$ is calculated for each node *i*.

(1) $$d_{w}=2*{\left[a*\left(x_{i}-x_{0}\right)\right]}^{2}+{\left[b*z_{i}\right]}^{2}$$

In this equation, *a* and *b* are the major and minor radii of the ellipse, as defined in Figure 1, and $x_{0}$ is the x-coordinate of the reference point. If the damage is considered to grow only from the top, the reference point is taken at the bottom of the weld. For a symmetrical damage growth pattern, the reference point is the centroid of the weld. The nodes with the highest weighted distance are then removed from the weld area to artificially introduce damage. The number of nodes that are removed increases from 10% to 90% of the initial amount.

### 2.4. Finite Element Analyses

This section contains the information regarding the setup of the FE models, such as mesh specifications, interface modelling, element types, and boundary conditions. The FE analyses were performed with Abaqus 2020 [35]. The analyses were used to enable the virtual testing of different spot weld shapes and sizes without the need to perform a large amount of expensive experimental testing. FE also allows more precise control of the shape and size without relying on manufacturing methods where an exact shape or size is not always achievable. The explicit solution method from Abaqus was used for better convergence. Both the adherends and the interface layer were assumed to be linear-elastic for the purpose of this study. The option APPLICATION = QUASI-STATIC was used, since this command chooses a method with very significant numerical damping. As such, the solver ensures that the static equilibrium does not evolve into a dynamic equilibrium and avoids the dominance of inertial forces. A mesh convergence check and a sensitivity study for the numerical parameters were performed; these results can be found in the online data repository [36].

#### 2.4.1. Mesh Parameters

For the adherends, 8-noded continuum shell elements (SC8R) were used. There were 2 elements through the thickness of each adherend, each 1.05 mm thick. Over the entire specimen, the element width was 0.5 mm. Figure 1 shows a shaded region of interest in the centre of the specimen. The element length outside of this region of interest was 2.0 mm. And the elements in the centre of the specimen had a length of 0.5 mm.

#### 2.4.2. Interface Specifications

**Figure 3:**
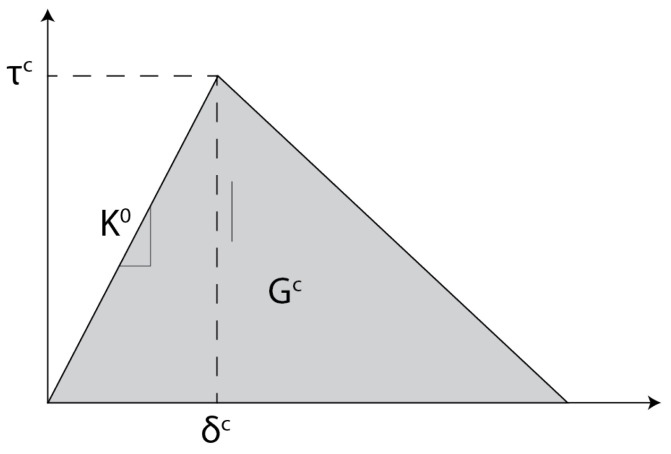
Bi-linear TSL for a Cohesive Zone Modelling (CZM) with the critical separation ($\delta^{c}$), critical traction ($\tau^{2}$), interfacial stiffness ($K^{0}$), and critical Strain Energy Release Rate (SERR) ($G^{c}$).

To determine the static failure displacement and corresponding load, the interface is modelled with the built-in cohesive elements using a bi-linear Traction-Separation Law (TSL) with the parameters shown in Table 3. An example of a bi-linear TSL with the numerical parameters is shown in Figure 3.

#### 2.4.3. Boundary Conditions

**Figure 4:**
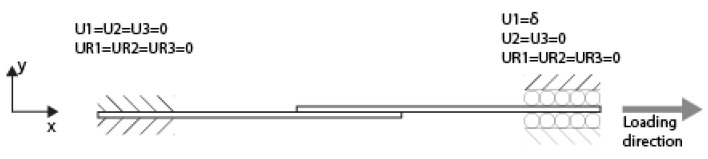
Boundary conditions for the FE analyses.

Figure 4 shows the boundary conditions applied on the hatched areas in Figure 1 in the FE analyses. On the left side, all six degrees of freedom were restricted. On the right side, the only unrestricted degree of freedom is the displacement along the x-axis. The loading was applied with a prescribed displacement in the positive x-direction. The boundary conditions at both edges are applied as nodal constraints on all the nodes within the hatched areas.

#### 2.4.4. Output Parameters

All the specimens were loaded until static failure of the specimen occurred. At this point, the reaction force at the loaded edge was obtained to compute the resulting compliance for each of the specimens.

## 3. Results

**Figure 5:**
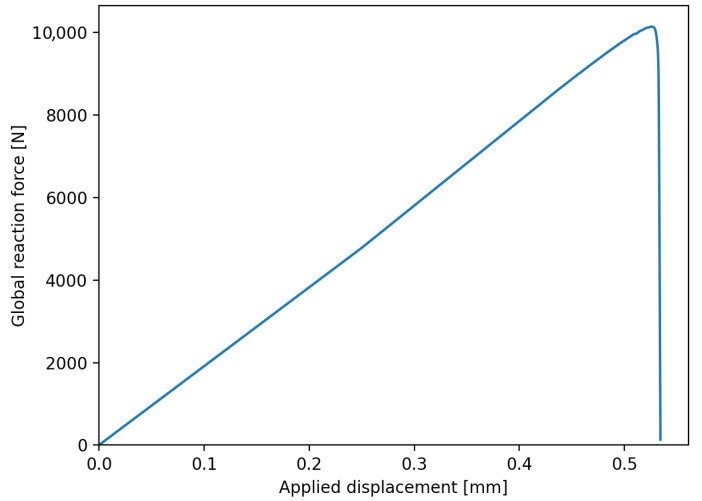
Applied displacement and reaction force for a spot weld with an area of 205 mm^2^ and an aspect ratio of 1.

As explained before, three aspects were varied between the different specimen configurations: total welded area, initial aspect ratio, and symmetric or asymmetric damage growth. The results are first shown for each aspect separately to show the influence of these aspects on the relationship between the weld area and the resulting compliance. As an example, Figure 5 shows the applied displacement and reaction force for the specimen with a spot weld with an area of 205 mm^2^ and an aspect ratio of 1. The compliance is calculated using the ratio of displacement and reaction force at the peak of this curve.

**Figure 6:**
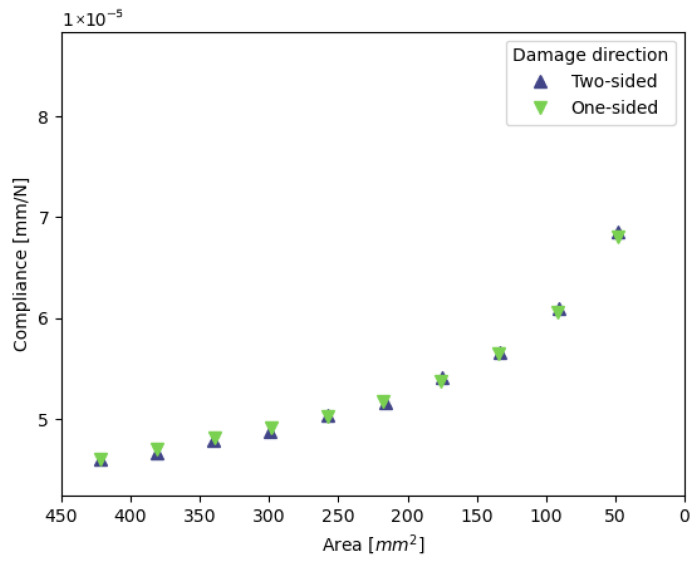
Relation between compliance and the remaining weld area for identical initial welds with a different growth direction. Data is shown for the specimens with blue symbols in Table 2.

Figure 6 shows the relationship between compliance and the remaining weld area of two different damage growth directions with the same initial welds. These results are obtained from the analyses with an initial aspect ratio of 1 and an initial size of 205 mm^2^. This subset is indicated with the blue symbols in the test matrix Table 2.

**Figure 7:**
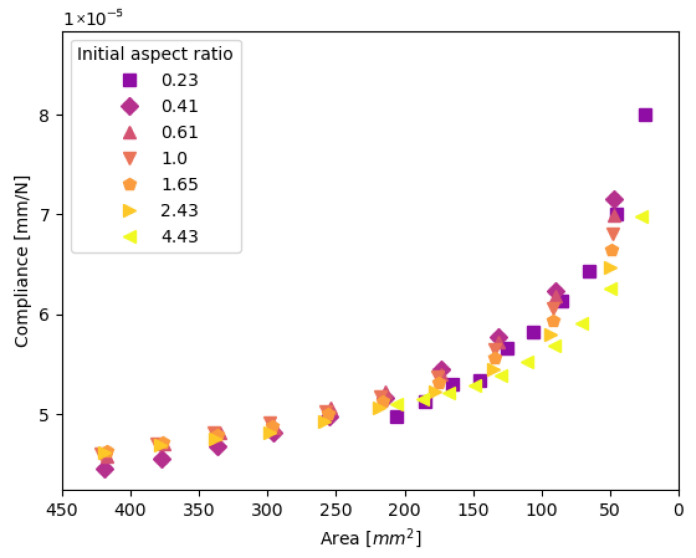
The relation between compliance and the remaining weld area for welds with different initial shapes. Data is shown for the specimens with orange symbols in Table 2.

In Figure 7, the resulting compliance is plotted for the specimens with an initial aspect ratio between 3.5/15.5 and 15.5/3.5, an initial size of 205 mm^2^, an initial aspect ratio between 7/17 and 17/7, and an initial size of 420 mm^2^. The evolution of damage is symmetrical from the top and bottom of the weld. This subset is indicated with the orange symbols in the test matrix Table 2.

**Figure 8:**
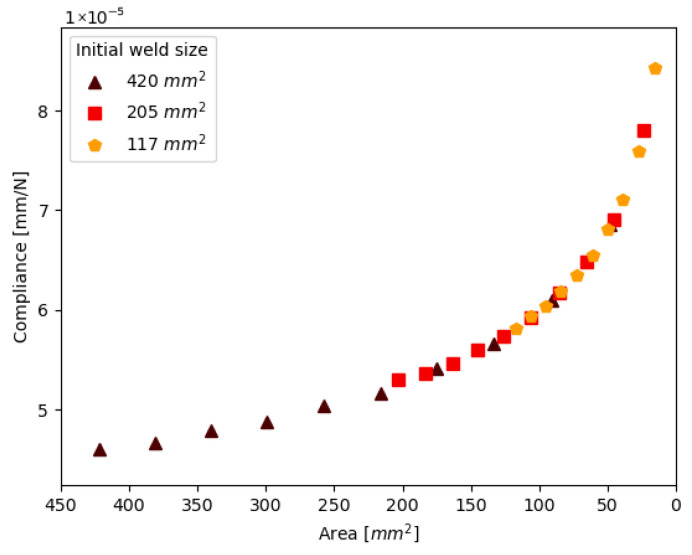
The relation between compliance and the remaining weld area for welds with different initial sizes. Data is shown for the specimens with green symbols in Table 2.

Lastly, the results are plotted for welds with the same shape and growth direction but a different initial weld size. These results are presented in Figure 8. The initial welds were all circular welds with symmetric growth of damage from the top and bottom. The green symbols in the test matrix Table 2 indicate the specimens used in this graph.

## 4. Discussion

**Figure 9:**
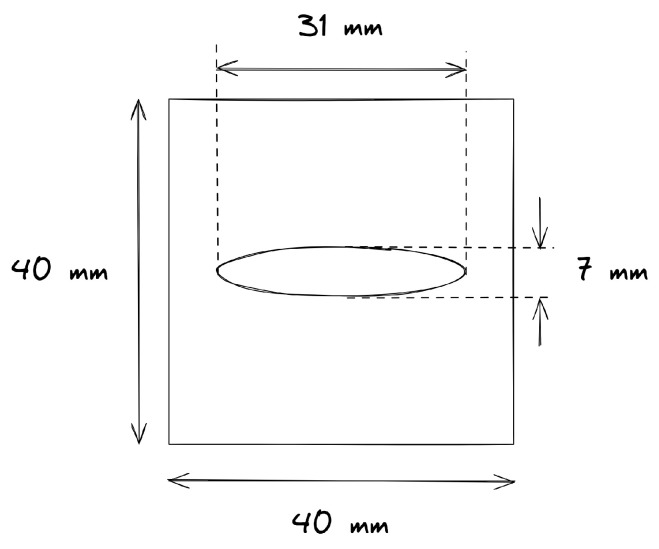
Illustration of the weld with aspect ratio 15.5/3.5.

**Figure 10:**
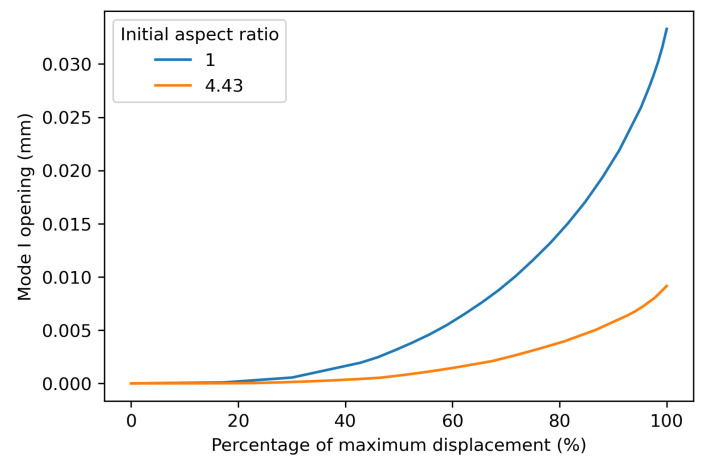
Mode I opening during the static FEA of the welds with an aspect ratio of 1 and 4.43, with a weld area of 205 mm^2^.

Figure 6, Figure 7 and Figure 8 show that there is very little influence of the weld shape and size on the relationship of its area with the resulting compliance. The only deviation can be seen in Figure 7, where the largest aspect ratio of 15.5/3.5 shows a different trend. The illustration in Figure 9 shows that this spot is nearly the entire overlap width and only spans a small part of the height of the overlap. Due to the high aspect ratio, there is only a small gap between the weld edge and the overlap edge in the transverse direction, which increases the specimen’s resistance to secondary bending and thus limits the peel stresses and mode I opening at the edge of the weld. Figure 10 shows the development of the mode I opening for the specimens with aspect ratios of 1 and 4.43 and a weld area of 205 mm^2^. These results are from the analyses where there is 0% damage, where the difference is the largest. Due to this difference in peel stress, it is expected that the correlation between the global compliance, the weld area, and the initial assumption does not hold.

**Figure 11:**
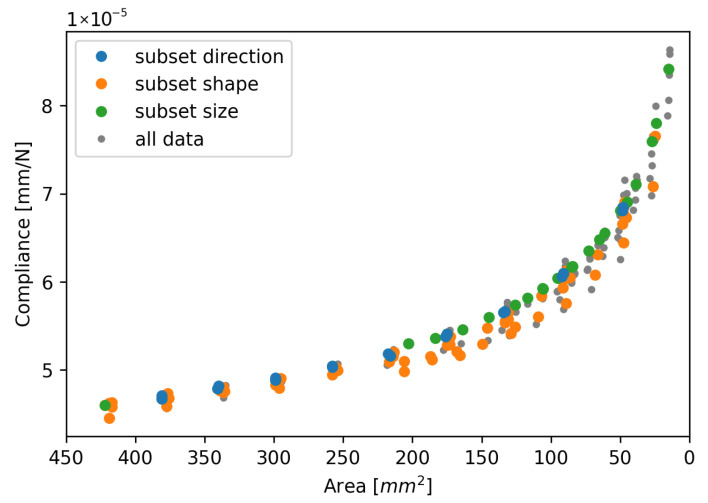
Compliance comparison for all specimens from Table 2.

Figure 11 shows the comparison between the compliance and the remaining weld area for all the different initial weld shapes and sizes, and the different growth directions as shown in the test matrix in Table 2. It can be seen that the results generally follow the same trend, regardless of the initial conditions and the shape evolution.

### Experimental Validation

The results from the FE analyses were compared to previously published experimental data [3]. In this work, fatigue testing was performed on seven different SLS specimens with an ultrasonic spot weld. DIC was used to monitor the specimen during the experiment, and the relative displacement between two points was extracted to obtain a virtual extensometer measurement. The compliance was then computed by dividing this displacement by the measured reaction force from the testing machine. The DIC also captured the surface strain, which was used to determine the state of the spot weld and compute the area through the experiment. The manufacturing specifications, loading parameters, and further information regarding data collection and post-processing are presented in the publication by Smeets et al. [3]. The experimental data is available in [37].

**Figure 12:**
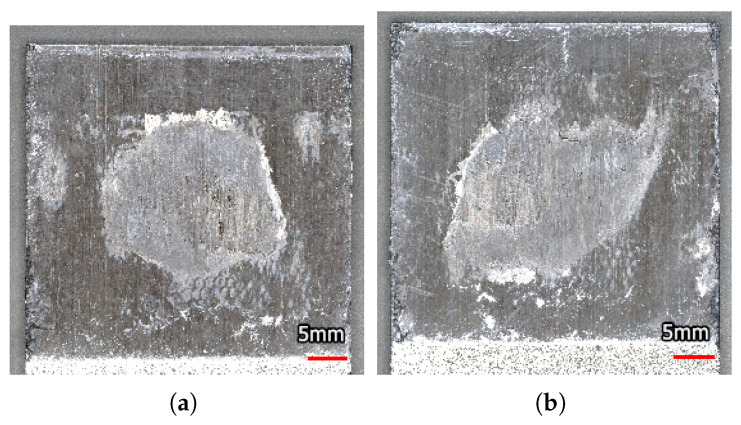
The fracture surface of two of the specimens shown in Figure 13, taken from Smeets et al. [3]. (**a**) Regular weld shape. (**b**) Irregular weld shape.

The data of two of the specimens were used to compare with the current numerical results. The fracture surfaces of these specimens are shown in Figure 12. These fracture surfaces were obtained with the Keyence VR-5000 microscope [38].

**Figure 13:**
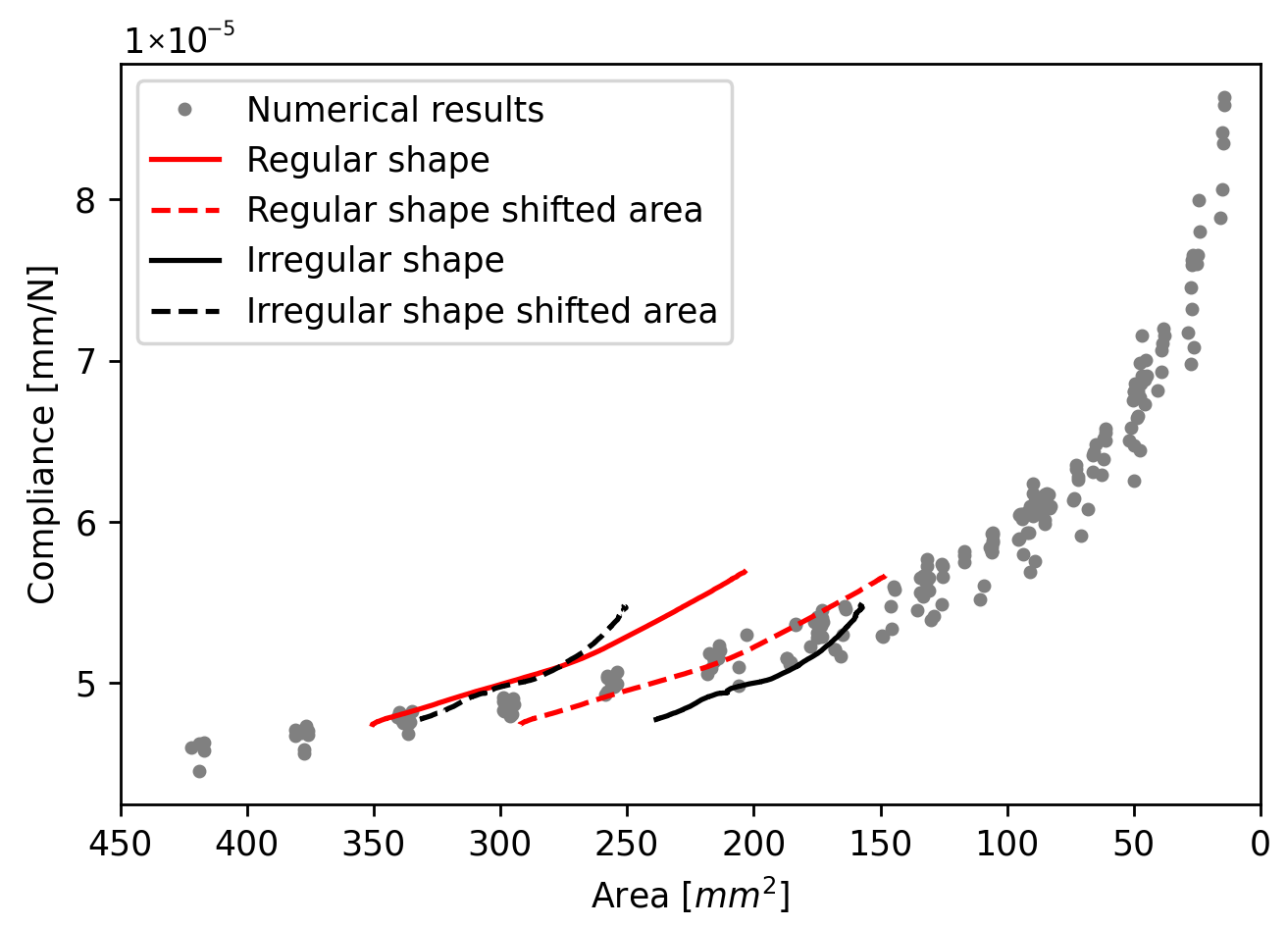
Relation between compliance and remaining weld area for all specimens combined with experimental data from Smeets et al. [3] with a regularly and irregularly shaped spot weld, including results with a correction for the initial area.

In Figure 13, the experimental data from the study by Smeets et al. [3] is presented with the data from this study. The continuous lines show the experimental data for a weld with a regular shape and one with an irregular shape. Both welds can be seen in Figure 12. To obtain continuous functions, the experimental data were smoothed using a half-cosine function as presented by [39]. Smeets et al. concluded that the measurement method reliably shows the progression of the damage throughout the fatigue life; however, the exact area might be misrepresented. To correct for this, the area of each post-mortem fracture surface was measured, and the experimental results were corrected such that the area measured on the pristine specimen at the start of the experiment corresponds to the post-mortem fracture surface. This causes a horizontal shift in the graph. The dashed lines represent the corrected experimental data. This shows that there is a strong match between the trends observed in the numerical and experimental data, confirming the insignificance of the shape of the spot weld when considering the relationship between its area and the resulting compliance. It should be noted that the results of the specimen with the irregular weld shape show more deviation from the numerical results. The FEA has the advantage that there is control over the exact shape of the interface. This also means that imperfections only exist when they are deliberately added. Therefore, there is no scatter in the numerical data due to imperfections in the specimens, the loading, or the environment. In experimental tests, there is a lot less control over both the interface and the testing conditions. Due to inherent variation in the produced welds, there is also a variation in the resulting trends. If this variation were included in the FEA, the numerical results would show a scatter band. The regular-shaped weld is more in line with the perfect shapes from the FEA; therefore, it is to be expected that the results align better.

There are some important remarks regarding the results. Firstly, the goal was to investigate whether the relationship between global compliance and weld area is sensitive to the shape of the spot weld. There are numerous factors that will affect the global compliance of the specimens. Some of these factors mainly affect the strength of the material, such as the Heat Affected Zone (HAZ), which is created during the welding process and locally changes the crystallinity of the matrix material [40] or other local defects. However, these phenomena do not influence the Young’s modulus of the material significantly; thus, their effect on the compliance will be limited. Other factors have a larger effect on the specimen stiffness, such as the material of the adherends, the adherend length, and the thickness. A significant change in specimen compliance will result in a different relationship between the weld area and the compliance, but the relationship will still be insensitive to the shape of the weld. In other words, the lines in Figure 13 will be translated or follow a different trend, but there will still be a trend that does not depend on the spot shape, and the conclusions drawn in this paper will still hold. Furthermore, the current study only includes shapes that are based on the previous experimental observations. In order to develop a more robust prediction model that is applicable not only to circular spot welds but also to other shapes, such as continuous or rectangular welded interfaces, a wider range of shapes should be investigated.

## 5. Conclusions

The aim was to investigate the influence of the size and shape of a spot weld on the relationship between the remaining weld area and the global compliance in an SLS specimen using FE analyses. The results showed no significant influence of the weld shape or size in any of the cases except for spot welds with an aspect ratio over 4, which is outside the expected range in manufacturing. Therefore, it is concluded that knowing the exact shape of a spot weld is not needed to assess its state based on global compliance when the weld is within the expected range of aspect ratios.

These results show that an initial quality assurance is important to ensure that the irregularity does not exceed acceptable limits. However, there is no need to continuously monitor the shape evolution during mechanical testing to gain insight into the damage state. This will greatly simplify the necessary equipment and data processing for mechanical testing, therefore increasing the potential for performing experiments with different configurations. This will allow for more investigations regarding the fatigue performance of ultrasonic spot-welded joints and how the performance is influenced by aspects such as the processing parameters, adherend properties, and weld shape.

The study aimed to investigate the limits of the hypothesis that was made based on previous experimental testing. An investigation into the relationship between compliance and the area of the weld was presented in this study. To expand our understanding of the underlying phenomena and further determine the range of applicability, future studies should focus on investigating the mechanisms that play a role in the influence of the spot shape and quantifying the effect of specimen compliance. This knowledge would allow the development of damage progression models and fatigue damage prediction based on the weld area and the specimen compliance.

In applications outside of a research context, ultrasonic spot welds are rarely applied as single-spot joints; multi-spot configurations are most often used. These types of joints are subjected to more complex loading scenarios, since the load transfer through each spot will not be constant. Therefore, these findings cannot simply be applied to multi-spot joints. When a spot weld in a multi-spot configuration is damaged, the load is redistributed to the neighbouring spots, which means the global compliance does not change significantly. However, when the damaged spot is located on the outer row of the overlap, the secondary bending of the joint will change during the damage process, changing the compliance. Overall, in a multi-spot configuration, the relationship between the accumulated damage and the global compliance will be different from that for a single-spot configuration, but it is expected that this correlation will also be insensitive to the shape of the individual spots. Further research is needed to investigate how the arrangement of a multi-spot configuration and the shapes of the spots within such a configuration influence mechanical performance and the relation between the total joint area and global compliance.

The potential impact of these findings is twofold; on the one hand, the reduced need for complex measurement techniques during experimental testing campaigns means that both testing and data processing can be faster and less costly. This will accelerate the development and characterisation of the ultrasonic welding process, which will extend the range of possible applications and lead to weight savings in load-carrying structures in the aviation sector.

On the other hand, these conclusions can also have implications in other fields. For example, planar delaminations can be considered the inverse of a spot weld, where the entire interface remains intact, except for a smaller, often circular area in the middle of the interface. A similar analogy can be drawn when considering distributed cracks in metal materials. This work shows that in both of these cases, the area of the feature is a much more significant parameter than the shape or characteristic length, again resulting in a less complex experimental testing procedure and thus creating potential for faster innovations.

## Figures and Tables

**Table 1:** Standard modulus carbon UD tape material properties from the manufacturer’s datasheet [34].

Parameter [Unit]	Value
$E_{xx}$ [GPa]	135
$E_{yy}$ [GPa]	10
$E_{xy}$ [GPa]	4.3
$\sigma_{max,xx}$ [MPa]	2410
$\sigma_{max,yy}$ [MPa]	86
$\sigma_{max,xy}$ [MPa]	152
ν [−]	0.33
lay-up	${\left[0/135/90/45/135\right]}_{s}$

**Table 3:** Cohesive interface parameters, based on [34].

Parameter [Unit]	Value
K^0^ [Nmm3]	$1\times 10^{6}$
$\tau_{I}^{c}$ [MPa]	120
$\tau_{II}^{c}$ = $\tau_{III}^{c}$ [MPa]	60
$G_{I}^{c}$ [kJm2]	2.1
$G_{II}^{c}$ [kJm2]	2.6
Mixed-mode criterion	BK
Mixed-mode power η	1.62

## Data Availability

The original contributions presented in this study are included in the article. The data related to this paper is accessible through the 4TU.ResearchData repository at [36].

## Abbreviations

The following abbreviations are used in this manuscript:

CZM	Cohesive Zone Modelling
DIC	Digital Image Correlation
FE	Finite Element
FEA	Finite Element Analysis
FRP	Fibre Reinforced Polymer
HAZ	Heat Affected Zone
LMPAEK	Low Melt Polyaryletherketone
SERR	Strain Energy Release Rate
SLS	Single-Lap Shear
TSL	Traction-Separation Law
USW	Ultrasonic Welding
